# Researches Respecting Animal Heat

**Published:** 1820-05

**Authors:** James Down


FOR THE LONDON MEDICAL AND PHYSICAL JOURNAL.

### *Researches respecting Animal Heat.* By James Down, Esq.

In a paper written by me on the subject of animal heat, inserted in your Journal in September last, I observed in the postcript, that Æmilius's questions were not wholly answered; nor could they, without entering into an enquiry why animals with a double circulation are capable of existing in a very high range of temperature.

It has been observed by physiologists, that animals with a double circulation, and the other mammalia class, are found to live in any range of temperature our atmosphere produces, even from the polar circle to the torrid zone, without increasing or diminishing the heat of their bodies; therefore, the healthy standard of our heat appears to be at about ninety-six degrees Fahrenheit's scale. It has also been proved by experiments, that the human body can exist with impunity in an atmosphere far hotter than any found in a natural state upon our globe, and by far exceeding the boiling point, or 212; and this also without increasing the heat of the body immersed. It should be here observed, that the surrounding medium must be of the gaseous kind, which does not infringe upon the body, nor readily give off caloric, so as to produce injury upon the skin.

I trust that I have rationally accounted for the generation of animal heat, in the paper before alluded to, as my subsequent experiments have further proved the accuracy of my former opinions and observations therein contained.

In the early part of my experiments, I was particularly inconvenienced in the hot room where I performed them, by having the cement melted that connected the brass caps with the glass pneumatic apparatus which I used in breathing, to ascertain the quantity of air consumed by the lungs in a given time.

I was therefore under the necessity of having recourse to the expedient of lapping the brass or glass with loose tow, and then keeping it constantly wet with water: by this means I not only preserved the cement from melting, but rendered the metal and glass comfortable to the touch, which had before, when used without caution, blistered my lips and taken off the skin. This induced me to institute the following experiments, to see how far I could prove the evaporation from the surface of the body to be the principal refrigerant, when exposed to a higher range of the temperature than that of the body immersed.

In order to ascertain this point by experiment, I placed two thermometers with Fahrenheit's scale on a board, within about nine inches of each other; the bulb of one lapped with loose tow, which was afterwards moistened with sulphuric ether. I then entered the room with them, which was previously heated by a stove. The thermometer unlapped, which I consider gave the real temperature of the room, rose in about fifteen minutes to 120; whilst that lapped with tow and moistened with ether, sunk to 50. I afterwards entered the room with these thermometers prepared in the same manner, only the bulb of one that was lapped with tow moistened with rectified spirit of wine, and the other bare, which I also consider gave the temperature of the room. The one bare, or unlapped, soon rose to 132; and the other, which was moistened with spirit, stood at 84. I withdrew these thus, and prepared them in the same manner as they were in the previous experiments, only moistening the one lapped with tow with cold water, and then entering the room as before. The one bare, giving the temperature of the room, rose to 150; whilst the other stood at 95. To place this beyond a doubt, that it was the evaporation which was the refrigerant power, I prepared the two thermometers as before stated: I then entered the room with them, and, on the stove which heated the room, I boiled some water, into which I immersed the thermometer lapped with tow, which immediately rose to 212, or the boiling point. I then placed it by the side of the other, which stood at this time at 150. In the space of about half-an-hour, the thermometer that was dipped into the boiling water sunk to 98, whilst that unlapped continued at 150.

We find by these experiments, that evaporation from the surface of unorganized bodies is a sufficient refrigerant to prevent substances from obtaining a greater degree of heat than that of 98, which is about the heat of our bodies under the same circumstances.

In objection to evaporation being the refrigerant of animals, some physiologists have stated that animals which do not perspire, and consequently no evaporation takes place upon the surface of the body in a natural state, will resist the power of heat equally as well as those that do perspire; therefore, they say, evaporation cannot be the refrigerant.

This appears, on the first view, to be a formidable argument against my experiments, and it would appear that evaporation is not the refrigerant which preserves this class of animals, and renders them capable of resisting heat, and prevents their bodies acquiring a degree much above that of 98. By a little reflection, and a few observations upon this class of animals, we find that they copiously perspire and evaporate much humidity from the lungs; and the surface of this organ, exposed to the air, is far more extensive than that of the whole body. We thus find the refrigerant organ of these animals to be the lungs; but, to allow this class of animals to be as capable of resisting the same degree of heat as those that perspire by the skin with as little inconvenience, is what I cannot accede to; for, in the first place, the stimulus of heat will propel the circulating fluids to the evaporating surface: we therefore observe this class of animals, when they have taken violent exercise, or have been exposed to a greater degree of heat than their own bodies, to be much agitated and inconvenienced, the blood repelled from the skin circulates internally, their mouths are opened, their tongues suspended therefrom, and there is a violent action of the heart and internal arteries. The canine species of this class are particularly sensible how much evaporation from the skin assists the refrigerant power of the lungs, by immediately running into water after violent exercise in hunting in warm seasons.

The refrigerant of all animals is the evaporation from the lungs or skin, and they are calculated to bear heat with impunity, according to the surrounding medium in which they are immersed. Thus, a human being may enter a room, surrounding himself with an atmosphere or a gaseous fluid which is heated to 212, or more, with little inconvenience: the evaporation in this case rapidly takes place from the skin, and thus keeps the heat of the body at about 98. Whereas, one of the canine species that perspires by the lungs, bears this degree of heat with great inconvenience; but, at the same time, it is known that the human species can bear but a few degrees of heat above the natural temperature of their bodies when immersed in a more dense medium, as that of hot water. Thus, a hot bath, when used much above 100, becomes particularly disagreeable, and causes an uneasiness of the body which can scarcely be borne. This, on a superficial view, would appear to arise from the water infringing upon the skin, and being more ready to part with its caloric than air; and this certainly is the cause of water scalding, and producing an injury upon the body: but this evidently is not the mean of rendering a too-hot bath uncomfortable; for, though a hot bath at 110

may be uncomfortable to the whole body when immersed, yet any small part of the body may be immersed in hot water of this temperature without any disagreeable sensation. When the body is surrounded with hot water, (we will say, for instance, at 110,) the circulating fluids are brought to the surface of the body by the stimulus of heat in a greater proportion than in a lower range of temperature, we will say 90. This high temperature causes the perspiration to exude through the skin in a more copious manner than it does in a natural state; and this is what nature, in its ordinary operations, has recourse to for relieving itself of the redundant quantity of caloric; but in this case we shall find it will fail to relieve the body of heat or caloric, though the perspiration exudes through the skin in a copious manner: it happens at this time that the refrigerant organ, the skin, is in close contact with the surrounding water, and consequently no evaporation can take place from the surface, as it does in the atmosphere; therefore the refrigerant power of evaporation by the skin ceases, and the only refrigerant we have left under these circumstances is evaporation by the lungs, and this must be very limited in the human body. I am of opinion, therefore, that the temperature of the human body under these circumstances may be considerably raised above that of the natural standard, though I have no disposition to subject myself to the experiment; well knowing that a hot bath of 110 or 120 is more intolerable than an atmosphere heated to 212: and this does not arise in consequence of the water being more ready to impart its caloric than that of air, but in consequence of the refrigerant power of perspiration ceasing, evaporation being prevented by the surrounding water.

The canine species, and those animals which perspire by the lungs, are more capable of resisting heat given by a hot bath than those animals are which perspire by the skin, for this obvious reason: their refrigerant organ, the lungs, are left to perspire and evaporate in their natural state; and this keeps the body nearly in an equal state of temperature, having free liberty to evolve the caloric absorbed from the hot water.

From what has been observed, it will appear, that those animals perspiring by the skin are capable of bearing the greatest degree of heat in an atmosphere where evaporation can be carried on. On the other hand, those animals that perspire by the lungs are capable of resisting the greatest degree of heat given off by a hot bath, the hot bath not interfering with their refrigerant organ, the lungs.

All that I have said upon this subject will amount to nothing, and appear fallacious, unless I remove an error that has crept into physiology; and to do this I must play the critic. I am not willing to enter upon this subject, knowing my incompetency; and at the same time I am well acquainted with the gentleman's abilities as a physiologist: therefore I would not, in this instance, attempt to remove any one's errors, if they were not actually in my way.

Richerand informs us, that "fishes and frogs have been known to live and retain their temperature in mineral waters nearly of a boiling heat." He was led to this by Sonerat's voyage to the East Indies; and he afterwards tried some experiments, to prove the accuracy of the statement. This is an error that blocks up the avenues of truth, and, were I to pass by it unobserved, I should leave in my rear a fortress, that a standard might be rose upon to batter down all that has been said upon the subject of animal heat. It is not very astonishing that a traveller should relate some marvellous stories; nor is it astonishing, that some people are found credulous enough to believe them. This eminent physiologist, bewildered as he was upon the subject of animal heat, half believed these tales, and instituted some experiments to prove the facts; and I have no doubt but he proved them to his own satisfaction. But the manner in which they were performed is not satisfactory to me; and it is so obvious an error, that I shall not give myself any trouble to try experiments to contradict it. Lest my readers may think me too confident in my assertion, and this too against experiments, I shall notice some tried by a gentleman not interested on this subject; and they seem to be so faithfully performed, that I would sooner depend upon them than any of my own, as I might possibly be biassed by opinion. These experiments were performed by Mr. Wilford on the boa constrictor, the largest of the serpent species, and are to be found in the Journal of Science and the Arts, No. xi. page 115. We here find a plan of his experiments, which consists of a table ruled into six columns: the first, for the month; the second, the day of the month; the third, the hour of the day; the fourth, the temperature of the atmosphere; the fifth, the temperature of the snake; the sixth, for observations. The whole of these experiments consist of thirty-nine; they were began on the 28th of October, 1815, and ended on the 5th of June, 1816. In most of these experiments there was not more than a degree, and in some not more than half or a quarter of a degree, difference in the heat of this animal, than that of the atmospheric air: only in two instances did the snake exceed the atmosphere in heat three degrees, and in one instance did the atmosphere exceed the snake two degrees.

Long before I read these experiments, the inference I had drawn was, that animals with a single circulation, such as fishes, frogs, reptiles, &c. had no means of regulating the temperature of their bodies, but were dependant upon the surrounding medium for the heat they possess. In the first place, they support life with but little consumption of air; this is a barrier to the generation of heat: and, in the next place, they do not perspire; therefore they have no refrigerant power.

Richerand further states, that evaporation from the surface of the body does not satisfactorily account for the refrigerant power of animals; "for the evaporation of the fluids contained in dead animal substances does not prevent their being roasted on the application of heat." It is true, that a piece of meat may be roasted by the side of a human being, and his or her temperature may not exceed that of 98; but it does not follow that evaporation is not the refrigerant, because the meat is roasted by the application of heat. In the first place, we must examine what the living human subject is; and, in the second, compare it with the dead animal matter. The living human subject consists of solids, with a vast quantity of fluids, with tubes conveying these fluids from the centre to the surface, and terminating or emptying themselves into an innumerable quantity of capillary cutaneous vessels, which secrete or separate from the blood the watery parts; and these are connected with ducts, that pour it out upon the skin, which will thus be kept constantly wet, that evaporation may not be impeded. The dead animal substance has not the same advantage for evaporation. In the first place, there is not the same quantity of fluids in proportion to the size of the substance, as there is in the living animal; and, in the second, it has no tubes circulating the fluids from the centre to the surface; and, thirdly, no cutaneous capillary vessels to separate the watery fluids; and, fourthly, no skin to pour these fluids upon. If we place the dead animal substance in the same advantageous situation for evaporation, we can as easily protect it from being roasted as that of the living animal. The manner in which I shall propose to accomplish this will be, to wrap the meat in a cloth, and keep it constantly wet with warm water at about 96. Under these circumstances, the dead animal substance is placed with equal advantage as that of the living animal for evaporation; and we can no more roast the one than the other, by the application of any degree of heat usually had recourse to in heated rooms for the purpose of experiments. This I have before evinced by the experiment of lapping the bulb of the thermometer in loose tow, and keeping it wet with water.

What I have usually observed to have taken place when I have entered heated rooms for the purpose of experiments, is, that my pulse has risen from about 80 strokes in a minute to 130; a profuse perspiration has taken place, apparently issuing from every pore of the body, and but little consumption of atmospheric air. But, to ascertain the quantity, I was much perplexed in rooms of a high temperature; for the heating or cooling of confined portions of air in the pneumatic apparatus, appeared to increase or diminish the quantity, when no increase or decrease had actually taken place. To do away with this inconvenience, I placed the apparatus out of the room, connected a pipe with them, which I conducted through the wall into the room, so as to enable me to breathe into it. But I found this also attended with so much inconvenience to prove the quantity of air consumed by the lungs in a given time, that I was induced to abandon the heated room, and to have recourse to colder air to ascertain this point.

I have frequently exposed my body for a length of time without a coat, in a frosty air, when the thermometer has stood many degrees below the freezing-point, and then breathed confined portions of atmospheric air; and soon after entering a room heated to 56 or 60, and then also breathed confined portions of atmospheric air. Under these and similar circumstances, I have constantly observed the quantity of oxygen absorbed to be in proportion to the heat evolved by the body, which may be occasioned by a cold surrounding medium, exercise, or any other means that promotes perspiration; and am of opinion this universally takes place in the healthy action of the lungs.

It is not to my present purpose to enquire the cause of this sympathetic action between the lungs and the skin, as it will suffice, in this case, to state the facts, and admire the wisdom of our Creator; for, without this sympathy, man could not exist in the vicissitudes of heat and cold which our atmosphere is constantly undergoing. When this sympathy is disturbed, even for a short space of time, we see to what distress and misery man is subject; as this appears to be the real, the much-sought-for cause, of spasmodic asthma.

It has been known to physiologists for a length of time, that, if the arterial blood escaped from the arteries into any part of the body, this blood would become carbonized or venous. To put this question beyond doubt, Mr. Hunter tried the experiment upon a dog. He tied the femoral artery in two places, so as to confine a portion of blood between the two ligatures: a short time afterwards he examined it, and found that it was changed from arterial to venous. This will account in some measure why the blood on the right side of the heart, or in the veins, should in a hot temperature, or the last portions of blood drawn from the arm, become nearly of the colour of arterial. If our bodies are immersed in a hot temperature, the stimulus of heat increases the action of the heart and arteries, and, consequently, the motion of blood in the arteries. The blood is also circulated more from the centre to the surface; secretion is partially stopped, only that of perspiration continues: the blood therefore passes quicker from the left to the right side of the heart, under these circumstances, than it does in the ordinary mode of circulation. This will also account for the last portions of blood drawn from the arm being more like arterial than that of the first. By abstracting blood from a vein, we lessen the quantity at the right side of the heart; the arteries and heart contract upon their volume, and propel the blood into the veins to a certain degree: but, when this contraction no longer takes place, syncope ensues. Thus it is that the latter portions of blood are not acted upon by the glands, and pass quicker from the left to the right side of the heart. I think this is an answer to Æmilius's questions, but it may not satisfy critical readers; for it will, probably, appear a paradox, that the blood should be more oxydated in a high temperature than it is in a low one, as the blood absorbs less oxygen in a high temperature than it does in a low one.

I shall account for this in two different ways: neither of them may be satisfactory, nor do I lay much stress or importance upon them. In a hot bath, or in high temperature, the blood is oxydated as usual at the expense of a little oxygen; it passes quickly from the left to the right side of the heart; secretion partially ceases, excepting that of perspiration, *condensation* of the blood not being necessary to keep up the heat, as the body at this time is really absorbing heat; the glands have but little action upon the blood, therefore only partially carbonize it. The blood, under these circumstances, has no strong affinity for oxygen in its passage through the lungs: hence it is that the lungs really do not absorb much oxygen in a hot temperature. Nor do the inhabitants of a hot climate consume as much oxygen as those of a cold one in their respiration; as their principal secretion is that of their cutaneous capillary vessels to separate the aqueous parts of the blood; but, in constantly throwing off the thin portions, it would become thick and grumous, and unfit for the office of vitality, were not some important eliminating gland to act upon the blood, decarbonize it, and throw off the necessary quantity of the solid portions: and this important gland I consider to be the liver. Hence it is that hepatic disease is most prevalent in a hot climate, and the pulmonary in a cold one. In a cold climate, the lungs have to absorb vast quantities of oxygen, to keep up the natural heat of the body. The liver, on the contrary, in a hot climate, has to decarbonize the blood, and eliminate the solid portions which cannot be held in solution by the urine, &c.

What has been said upon the liver my readers may consider hypothetical; and I grant it is, because it cannot be demonstrated by experiment; therefore it should be received with caution: but, would the limits of the Journal allow me to enter into the process of *assimilation* and *elimination*, I should not despair of proving the accuracy of the opinion by arguments founded on anatomy and chemistry.

*Leicester; March* 15, 1820.

